# Associations between indoor relative humidity and global COVID-19 outcomes

**DOI:** 10.1098/rsif.2021.0865

**Published:** 2022-11-16

**Authors:** C. A. Verheyen, L. Bourouiba

**Affiliations:** ^1^ Harvard-MIT Health Sciences and Technology, Boston, MA, USA; ^2^ Institute for Medical Engineering and Science, MIT, Cambridge, MA, USA; ^3^ The Fluid Dynamics of Disease Transmission Laboratory, MIT, Cambridge, MA, USA

**Keywords:** SARS-CoV-2, epidemiology, indoor relative humidity, weather, climate, respiratory viral outbreaks

## Abstract

Globally, the spread and severity of COVID-19 have been distinctly non-uniform. Seasonality was suggested as a contributor to regional variability, but the relationship between weather and COVID-19 remains unclear and the focus of attention has been on outdoor conditions. Because humans spend most of their time indoors and because most transmission occurs indoors, we here, instead, investigate the hypothesis that *indoor* climate—particularly *indoor* relative humidity (RH)—may be the more relevant modulator of outbreaks. To study this association, we combined population-based COVID-19 statistics and meteorological measurements from 121 countries. We rigorously processed epidemiological data to reduce bias, then developed and experimentally validated a computational workflow to estimate indoor conditions based on outdoor weather data and standard indoor comfort conditions. Our comprehensive analysis shows robust and systematic relationships between regional outbreaks and indoor RH. In particular, we found intermediate RH (40–60%) to be robustly associated with better COVID-19 outbreak outcomes (versus RH < 40% or >60%). Together, these results suggest that indoor conditions, particularly indoor RH, modulate the spread and severity of COVID-19 outbreaks.

## 1. Introduction

The global coronavirus disease 2019 (COVID-19) outbreak, caused by severe acute respiratory syndrome coronavirus 2 (SARS-CoV-2), began at the end of 2019 and was declared a pandemic by the WHO in March 2020 [1]. As of October 2022, there have been more than 600 million cases and over 6.5 million deaths attributed to COVID-19 [2]. SARS-CoV-2 is largely transmitted through inhalation of virus-laden respiratory droplets emitted in a range of sizes and a range of exhalation turbulent gas puff clouds (e.g. breathing, talking, sneezing, coughing), with some remaining suspended in the air for extended periods of time [3–6].

Shortly after the pandemic was declared, experts began discussing the possibility of COVID-19 seasonality [7,8]. The association between outdoor weather and disease transmission has been the subject of debate, and a range of outdoor weather variables have been explored (e.g. temperature, enthalpy, absolute humidity or relative humidity) [8–12]. However, the mechanisms driving such cycles remain unclear, and contradictory findings on the role of humidity or temperature in viral outbreaks persist even for common diseases like influenza. Several human coronaviruses are endemic, often causing common colds during the winter timeframe; however, more severe coronaviruses like Middle East respiratory syndrome are not necessarily associated with seasonality [13]. Influenza is observed to cause cyclical epidemics [11,14,15]. These cycles occur in the temperate zones of the globe, where summer months tend to be warmer and wetter and winter months tend to be cooler and drier [16].

Studies seeking to link COVID-19 outbreaks and environmental conditions [17–21] most commonly focused on *outdoor* temperature, humidity and UV light. Overall, their conclusions varied considerably, with some reports describing clear links between COVID-19 and some outdoor weather variables, and others finding little to no evidence to support such a connection. The variation in results is likely due to differing data collection processes as well as differing statistical methodologies [17–19,22]. The timeframes of study and the geographical regions of study also varied from paper to paper, with some focusing only on initial outbreaks or only on individual countries. This could inadvertently lead to selection bias and reduce the generalizability of the results. Moreover, most studies focused on total confirmed cases, which can depend strongly on testing infrastructure, particularly early in the pandemic. Additionally, many studies used simple linear correlations, which can produce spurious results when computed on time-series displaying significant temporal trend (often present in cumulative case numbers). For studies with more advanced methodological approaches, the choice of a model's functional form, the handling of dataset outliers, the incorporation of non-meteorological factors, and the analysis of uncertainty in model inputs and model assumptions could all dramatically change the final conclusions [17–20]. Therefore, further analyses and robustness tests are urgently needed to confidently elucidate seasonal dependency of the SARS-CoV-2 outbreak. We proceed to do so in the remainder of this paper, starting by changing the focus from outdoor to indoor conditions.

Altered human behaviour (due to inhospitable outdoor conditions) is the most commonly provided mechanism for the relationship between seasonal weather and viral outbreak, yet this is perplexing when considering respiratory diseases. First, most societies now spend more than 90% of their time indoors (independent of external weather conditions) [23–25], so outbreak seasonality cannot be attributed entirely to seasonal human behaviours. Second, the majority of viral transmission occurs indoors rather than outdoors [26–28]. Third, the use of environmental control systems produces a divergence between outdoor and indoor environmental conditions [29,30]. Whether through natural gas, electricity or solid biomass fuels, people residing in cold regions dedicate a significant portion of their total energy expenditure to maintaining indoor comfort [31–34]. Therefore, it is not immediately clear how external weather variables could modify indoor viral spread, particularly without established biophysical mechanisms to support such a link.

**Figure 1.**
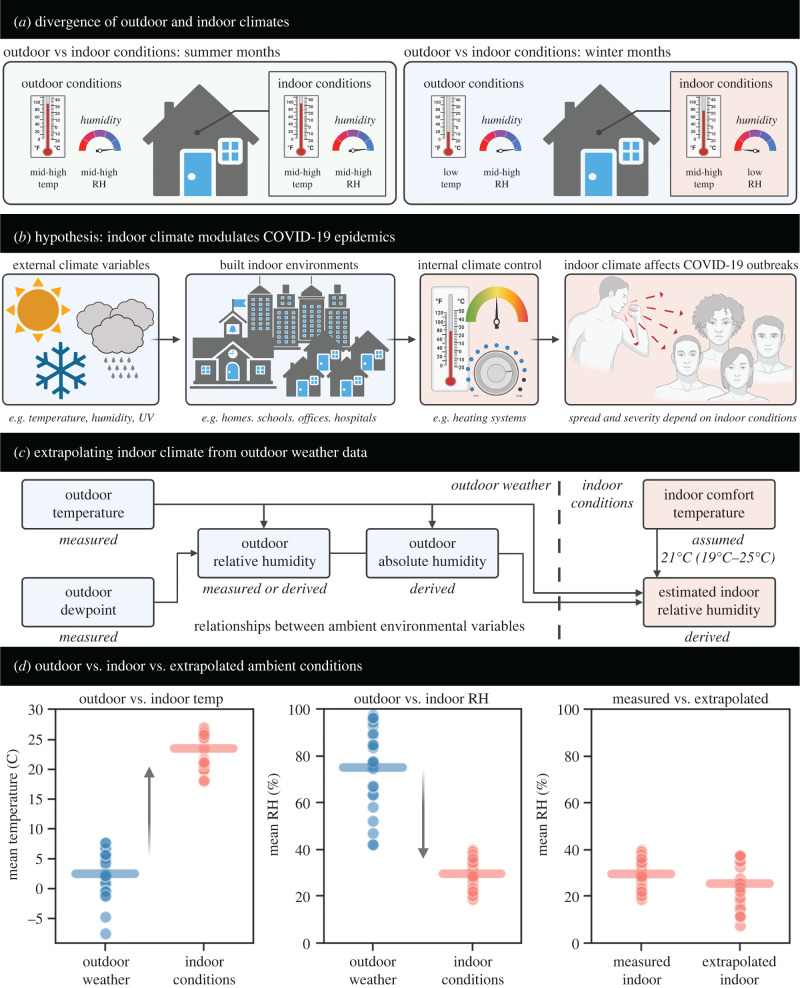
Graphical abstract, description, and experimental validation of indoor humidity estimation workflow. (*a*) Schematic illustrating the similarity in outdoor and indoor temperature and humidity conditions in summer, and the discrepancy in outdoor and indoor conditions in the winter. (*b*) Schematic illustrating the paper's driving thesis: outdoor weather variables change over time. Humans spend most of their time inside and can modify ambient conditions to satisfy comfort requirements; therefore, indoor rather than outdoor environmental conditions are most relevant to the evaluation of COVID-19's dependency on ambient conditions. (*c*) Schematic illustrating the computation of indoor environmental conditions. Outdoor temperature and dewpoint can be retrieved from global meteorological datasets and then used to derive outdoor relative humidity (RH) and absolute humidity. We validated this approach by comparing estimates to our direct experimental measurements of indoor versus outdoor RH and temperature for multiple types of indoor environments (see electronic supplementary material, figures S13–S20). With this validated approach, assuming occupants heat their indoor spaces to the human thermal comfort zone (21°C for main text results; 19–25°C in electronic supplementary material, figures S1–S6), we derive the estimated indoor RH from outdoor conditions. (*d*) The difference in outdoor and indoor temperature (left panel), outdoor and indoor RH (middle panel), and comparison of measured versus extrapolated indoor RH (right panel). The extrapolated indoor RH is computed as explained in (*c*). All three panels show pooled data from the literature collected in spring 2020, supplemented with data we directly experimentally collected in autumn and winter 2020–2021.

Conversely, cyclical variation in the indoor ambient environment may offer a mechanistic basis for the observed seasonality of respiratory diseases [35]. For example, *indoor* relative humidity (RH) has been shown to display a seasonal, cyclical pattern in temperate environmental zones (RH is a relative measurement representing the ratio of actual vapour pressure to saturation vapour pressure) [36,37]. In warmer months, the difference between outdoor temperature/humidity and indoor temperature/humidity is relatively small; however, during colder months, this discrepancy can become substantial (figure 1*a*). Naturally, humans prefer ambient conditions that maximize their comfort levels. This ‘human thermal comfort zone’ is defined as approximately 20–24°C during the winter timeframe [38,39], and therefore indoor spaces are typically heated to maintain comfortable ambient temperatures despite low outdoor temperatures. This heating process has the unintended consequence of drastically changing the RH. Cold air has a lower saturation vapour pressure than warm air, so outdoor absolute humidity (AH) levels are low in colder months even if outdoor RH is high (AH is a measure of the actual moisture content in the air). When cold external air is heated to comfortable levels, the RH plummets. Thus, occupants of heated indoor spaces likely experience low RH during the colder months in temperate regions of the globe.

Moreover, indoor RH is thought to be an important mediator of occupant health and disease [40–42]. It can affect both viral and host processes, ranging from respiratory viral stability and transmissibility to host immune function and mucous barrier integrity [43–45]. In particular, RH between about 40% and 60% has been discussed to be optimal for occupant health and to minimize the risk of disease transmission [46–48]. We hypothesized that indoor RH could have affected the global spread and severity of COVID-19 and may have been partially responsible for the observed regional heterogeneity in COVID-19 outcomes.

We proposed that *indoor, rather than outdoor,* environmental conditions are the relevant factors to consider when studying viral respiratory outbreaks (figure 1*b*). In this paper we investigate the potential relationship between *indoor conditions* and COVID-19 spread and severity, with the following focus questions:

— Are there regional differences in COVID-19 severity and epidemic dynamics?
— Can part of the regional variability in COVID-19 be related to *indoor relative humidity levels*?
— Is the association between indoor relative humidity and COVID-19 *robust* to alternative methodologies and confounding factors (e.g. outdoor weather variables or government intervention)?

We found that the answers to the three above questions are positive: regional COVID-19 outbreaks displayed a consistent relationship with indoor RH which is robust to alternative methodologies as well as varying outdoor weather conditions and government responses.

## 2. Results

We retrieved and processed COVID-19 statistics and weather data from 121 countries. Detailed methodology is provided after the main results. Key steps, assumptions and robustness checks are interwoven throughout the text. Given the substantial day-to-day variability in the data, all continuous variables were smoothed with a 7-day centred rolling average to reduce high-frequency noise while preserving the underlying signal (in electronic supplementary material, figures S40–S41, we quantify the consequences of alternative data processing strategies and show that our results are robust to this smoothing).

We partitioned the global dataset into Northern Hemisphere (NH) countries (latitude > 23.5°, *n* = 67), Southern Hemisphere (SH) countries (latitude < − 23.5°, *n* = 4) and Tropical (Trop) countries (23.5° > latitude > −23.5°, *n* = 50). We present most of the analysis in the main text through this regional lens. However, in electronic supplementary material (figures S30–S32) we performed the same analysis on the pooled global dataset and found the conclusions to be consistent. We also examined the effect of further regional stratifications by dividing the Tropics into northern and southern regions, and likewise we found the conclusions to be robust—see electronic supplementary material, figures S21–S23. The original data were retrieved and processed in mid-August 2020. To confirm our study's conclusions, follow-up validation datasets were retrieved in early December 2020 and late January 2021, and we find similar results. These are presented in electronic supplementary material, figures S33–S34. The choice of our data range was designed to capture pre-vaccine epidemic dynamics, closer to freely evolving outbreaks in fully susceptible populations. However, we also account for government-imposed mitigation strategies, such as isolation, quarantine and testing.

Others have argued that confirmed cases measure the spread of COVID-19, while confirmed deaths measure the severity of COVID-19 [49]. In the main body of this paper, we focus on death-related metrics due to the documented high variability of quantification of cases, particularly in the first part of 2020. However, in electronic supplementary material (figures S31–S32) we also analysed case-related results and show that both new cases of and deaths from COVID-19 display similar relationships across space and time with respect to indoor conditions. As discussed previously, COVID-19 outbreaks were non-randomly distributed around the globe with very different onset dates, which can introduce selection bias. To prevent such bias, we first applied a filter to isolate countries with more than 50 deaths, thus avoiding analysis of countries at the onset of their epidemics. We re-scaled each country's timeline, with epidemic onset time, t_0_, defined as the time when ≥5 confirmed deaths were reached. This allowed for a comparison of epidemic trajectories on a common timescale. Finally, we limited the analytical window to 0–120 days on the normalized timeline. This operation reduces the influence of countries with earlier outbreak onsets and ensures that countries with later epidemic onsets remain represented in the dataset on the common timescale. In electronic supplementary material, figures S37–S39, we quantify the effect of changing this analytical timeframe. Moreover, cumulative COVID-19 metrics increase over time and display obvious unsteadiness, which can lead to spurious correlations. To account for this, we applied serial differencing operations to the cumulative death counts. The first difference provides the daily deaths (new deaths), while the second difference provides the day-to-day difference in new deaths. Both metrics are dependent on the magnitude of the epidemic. Hence, as a third normalized metric we also examined the percent change in new deaths. The difference in new deaths measures the absolute change, while the percent change in new deaths measures the relative change, thereby allowing us to examine whether or not the magnitude of the COVID-19 outbreaks could change the study conclusions.

In order to compare COVID-19 outbreaks to ambient environmental conditions, we first retrieved global meteorological data and paired the spatio-temporal weather measurements to the corresponding date and latitude/longitude for each country's COVID-19 statistics. Outdoor temperature and dewpoint gave the outdoor RH and outdoor absolute humidity. Then, we computed the expected indoor RH based on the measured outdoor conditions and known and documented human thermal comfort (figure 1*c*), where cold outdoor conditions would require occupants to heat their indoor spaces to achieve a comfortable ambient temperature. For the figures presented in the main text, we take an indoor comfort temperature of 21°C, but in electronic supplementary material (figures S1–S6) we examined the robustness of all our results across a range of indoor comfort temperatures (19–25°C) and found our conclusions to be highly robust. Furthermore, to confirm the validity of our approach to computation of indoor RH, we analysed (1) previously published indoor measurements from three teaching hospitals in late March 2020 [50] and (2) our own experimental data collected from different types of buildings in autumn and winter 2020–2021. Figure 1*d* shows the results from the pooled data averaged over time. As expected, outdoor temperatures were very low while indoor temperatures were maintained at higher levels for human comfort. Predictably, the indoor RH was low even though the outdoor RH was high. At cold temperatures the outdoor saturation vapour pressure is low, so the absolute moisture content is minimal. If this cold, dry air is heated to comfortable temperatures, the saturation vapour pressure rises even if the absolute moisture content is unchanged, yielding a decrease in RH. When we compare the measured indoor RH to the extrapolated indoor RH we find very good agreement between the two, showing that, on average, our computational approach reliably captures indoor ambient conditions from outdoor conditions. We provide full results of the in-depth validation analysis in electronic supplementary material (figures S13–S20). Having established this methodology, we can now discuss the associations between indoor RH and COVID-19 severity.

### 2.1. Regional variation in ambient conditions and COVID-19 outbreaks

**Figure 2.**
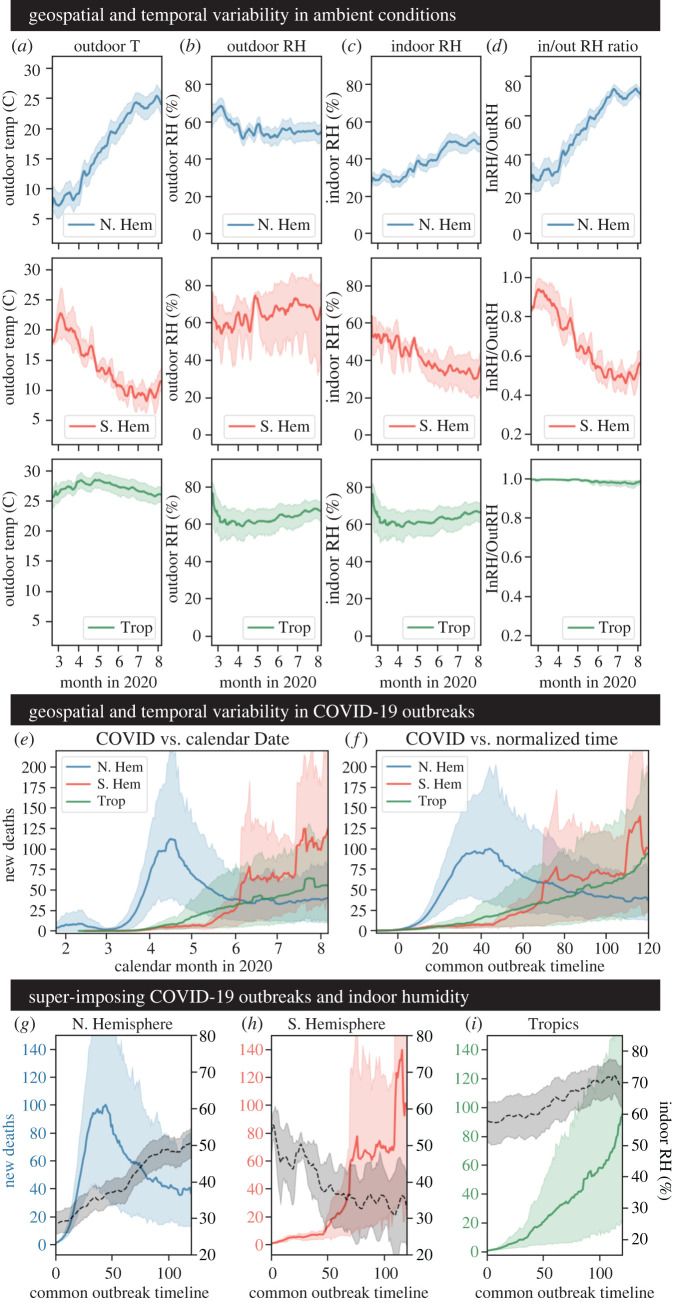
Geospatial and temporal variability in ambient conditions and COVID-19 outbreaks. (*a*) Outdoor temperature in degrees Celsius. (*b*) Outdoor RH in percent. (*c*) Indoor RH in percent. (*d*) The ratio of indoor to outdoor RH. Each metric was plotted against the 2020 calendar year. Daily measurements were grouped by region and averaged, and bootstrapped 95% confidence intervals were constructed for each estimate. (*e*) Daily confirmed COVID-19 deaths plotted against the calendar year in 2020. (*f*) Daily confirmed COVID-19 deaths plotted against the common timescale (where *t*_0_ is the timepoint at which a given country reaches or passes 5 cumulative confirmed COVID-19 deaths). Daily measurements were grouped by region, averaged, and bootstrapped 95% confidence intervals were constructed for each estimate. (*g–i*) The indoor RH in percent and the number of new confirmed COVID-19 deaths were both plotted against the common outbreak timescale (from *t*_0_ to *t*_120_) for the Northern Hemisphere countries (*g*), Southern Hemisphere countries (*h*) and Tropical countries (*i*). Daily measurements were grouped by region and averaged, and bootstrapped 95% confidence intervals were constructed for each estimate.

We first examined the average of the environmental conditions over time for each region. Figure 2*a* shows that, as expected, outdoor temperature increases in NH countries, while it decreases in SH countries as they move between seasons. Meanwhile, the temperature in Trop displays minimal change. The values for outdoor RH (figure 2*b*) are quite high year-round for all countries independent of geographical position (approx. 50% or higher for NH, SH and Trop locations). Indoor RH, however, differs across regions (figure 2*c*). For the NH, indoor RH is much lower than outdoor RH during the winter and early spring months (indoor RH < 40%, outdoor RH > 60%). However, as the spring season gave way to summer, the indoor RH approached the outdoor RH. The opposite trend is observed in the SH as it transitioned from similar indoor/outdoor conditions in summer to divergent conditions in winter (indoor RH < 40%, outdoor RH > 60%). Again, the Trop countries displayed remarkable stability in conditions over time. The discrepancy between indoor and outdoor environments in the temperate zone countries (NH, SH) became clear examining the ratio of indoor to outdoor RH over time (figure 2*d*). During the winter months, indoor RH was only a fraction of the outdoor RH (a result of the heating process). Thus, the occupants of these indoor spaces likely experienced dry ambient conditions.

Regarding average COVID-19 outbreak dynamics, we found a clear difference between the three geographical regions (figure 2*e*). For NH countries, COVID-19 deaths spiked in the March to May timeframe, before decreasing as spring gave way to summer. Conversely, for SH countries, COVID-19 deaths were relatively flat until spiking in the June to August timeframe (winter months in the SH). Lastly, COVID-19 deaths in the Trop were fairly consistent. The clear separation in outbreak curves persisted even after timescale normalization (to account for country-to-country variability in outbreak onset dates). On the common timeline, we still observed an early spike in the NH, a late spike in the SH, and a consistent upward trend in the Trop countries (figure 2*e*). The persistent separation of the regional outbreak curves confirms that there are true underlying differences in their epidemic dynamics.

When superimposing the temporal evolution of new COVID-19 deaths and indoor RH by region, new deaths increase rapidly in the NH in the first half of the common timescale, before decreasing gradually in the second half (figure 2*g*). In comparison, indoor humidity increases from low RH (<40%) in the first half to intermediate RH (40–60%) in the second half. In the SH, new deaths were low in the first half of the common timescale, before increasing rapidly in the second half (figure 2*h*). Here, indoor humidity decreased from intermediate RH in the first half to low RH in the second half of the common timescale. Finally, Trop countries displayed a consistent rise in new deaths across the entire common timescale, with a more rapid increase toward the end (figure 2*i*). Compared to the temperate regions, there was a smaller change in indoor environmental conditions, but there was a gradual shift from intermediate RH to high RH (greater than 60%). Taken together, these timeseries suggest that temperate climate zones (NH, SH) experience worse viral outbreaks during seasonal drops in indoor RH. Conversely, the tropical zones may actually experience increased viral outbreaks at high indoor RH.

### 2.2. Modelling COVID-19 outbreak metrics versus indoor relative humidity

**Figure 3.**
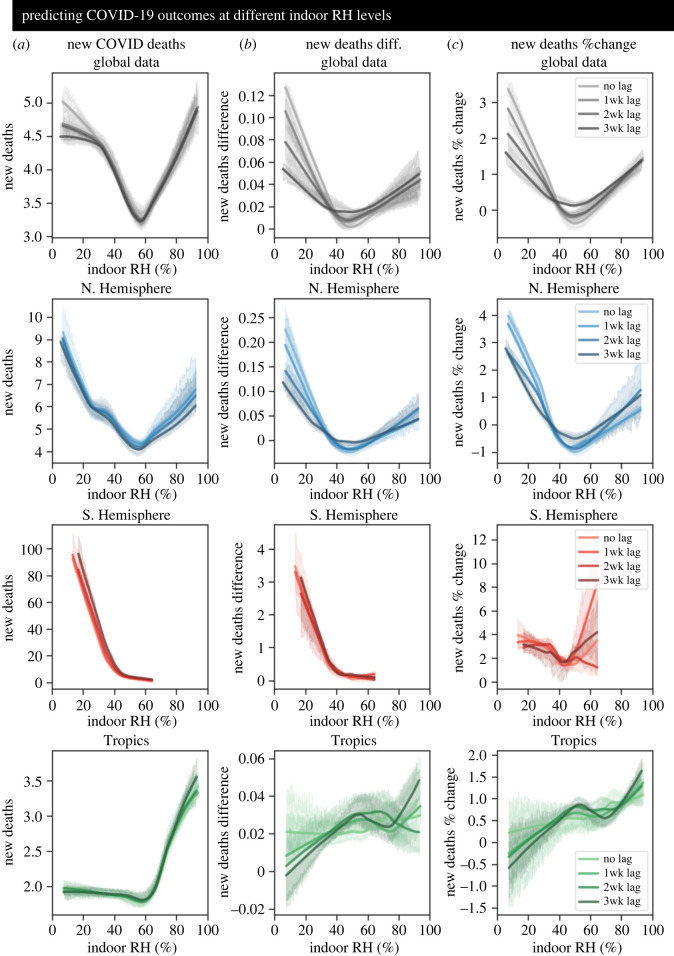
Dependency of COVID-19 on indoor RH. (*a*) Daily confirmed COVID-19 deaths, (*b*) day-to-day difference in daily confirmed COVID-19 deaths, and (*c*) day-to-day percent change in daily confirmed COVID-19 deaths were regressed on the indoor RH using a non-parametric LOWESS method to capture nonlinear structure without making assumptions about the model form. The resulting regression estimates were plotted across the range of data used for model fitting, and bootstrapped 95% confidence intervals were constructed for the estimates. To account for potential time delays between input and output, COVID-19 outbreak metrics were regressed on 0, 1, 2 and 3 weeks of time-lagged indoor RH. Models were created on the pooled global dataset (row 1) and on regionally stratified subsets representing the Northern Hemisphere (row 2), Southern Hemisphere (row 3) and the Tropics (row 4).

To more explicitly explore the link between indoor RH and COVID-19, we applied robust regression methods to model the underlying relationship without undue influence from outliers (additional information and sensitivity analyses can be found in electronic supplementary material, figures S35–S36). Given the potential for lagged dependencies, we fit the COVID-19 death-related statistics to indoor RH values with zero, one, two or three weeks of time lag. Initially, we employed an iteratively re-weighted least-squares approach with Huber's *T* weighting function to model the underlying dependency with a linear function (electronic supplementary material, figure S31). Generally, the temperate regions displayed a negative relationship between indoor RH and COVID-19, while the tropical regions displayed the opposite trend. Interestingly, the patterns were highly consistent across time lags. Viewed together, our robust linear modelling approach suggested that temperate zones and tropical zones may exhibit opposite associations between COVID-19 and ambient environmental conditions. However, we also considered that a linear model may not adequately capture the underlying structure in the dataset. A robust, non-parametric locally weighted scatterplot-smoothing (LOWESS) technique applied to the same data can flexibly fit nonlinear structure (figure 3*a–c*). Indeed, this approach revealed strongly nonlinear relationships between COVID-19 and indoor humidity. Global estimates for new deaths (figure 3*a*), difference in new deaths (figure 3*b*), and percent change in new deaths (figure 3*c*) were minimal at intermediate RH (between 40% and 60%). These estimates increased rapidly as indoor RH decreased below—or increased above—the intermediate range. The same general trend was mirrored by the NH subset, though the estimated COVID-19 statistics increased more gradually at the higher humidity values. Similarly, in the SH both the estimated new deaths and difference in new deaths displayed sharp increases as the indoor RH decreased below intermediate RH. Around intermediate RH, the outbreak metrics were minimal and quite consistent. For the percent change in new deaths, there was a moderate increase as the indoor RH decreased below intermediate values. Above intermediate RH, there was wide variability in the predicted value, and the value changed dramatically according to the time lag, thereby preventing a clear conclusion. In the Trop countries, estimated new deaths were relatively flat across low and intermediate RH, but increased sharply as RH increased beyond the intermediate range. For the de-trended metrics, confidence intervals were larger: the difference in new deaths showed a slight (approx. linear) increase while the percent change showed a stronger (approx. linear) increase as indoor RH increased. Together, these results support the association between very low (<40%) or very high (>60%) indoor RH and worse COVID-19 outbreaks and further reveal that there is a potential minimization of COVID-19 severity at intermediate RH levels (40–60%).

### 2.3. Associations between COVID-19 outcomes and indoor RH exposure

**Figure 4.**
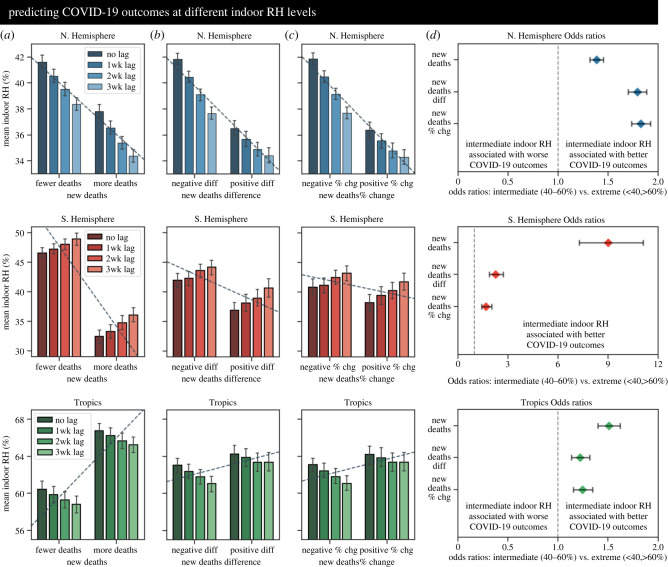
Intermediate indoor RH is significantly associated with better COVID-19 outcomes. (*a–c*) Comparison of mean indoor RH values for COVID-19 outcomes of differing severity. New COVID-19 deaths (*a*), the difference in new COVID-19 deaths (*b*), and the percent change in new COVID-19 deaths (*c*) were binarized into approximately equally distributed quantiles (lower half = less severe outbreaks, upper half = more severe outbreaks). Datapoints were grouped by relative severity and the average indoor RH was computed (with 95% confidence intervals for each estimate). Dashed lines are included as a visual guide and represent best-fit lines (fitting estimated indoor conditions to binary outbreak labels). Average environmental condition estimates were computed for 0, 1, 2, and 3 weeks of time-lagged indoor RH. Estimates were created on regional subsets representing the Northern Hemisphere (row 1), Southern Hemisphere (row 2), and the Tropics (row 3). (*d*) Regional odds ratios. Odds ratios (ORs) and 95% confidence intervals were calculated based on binarized COVID-19 outcomes (more severe versus less severe, same data partitioning as in *a*). Specifically, ORs were computed by dividing the odds of a worse COVID-19 outcome given extreme RH exposure (less than 40% RH or greater than 60% RH) by the odds of a worse COVID-19 outcome given intermediate RH exposure (40% RH to 60% RH). For simplicity, the ORs here are common odds ratios summarizing the pooled effect across 0, 1, 2 or 3 weeks of time lag (full results in electronic supplementary material, figure S32). ORs were calculated for regionally stratified subsets representing the Northern Hemisphere (row 1), Southern Hemisphere (row 2) and Tropics (row 3).

We recognized that the considerable spread in COVID-19 data reduced the reliability of exact quantitative predictions. As such, we then examined the environmental conditions associated with ‘better’ or ‘worse’ COVID-19 outcomes in a case-control fashion. We discretized the continuous COVID-19 outbreak metrics by partitioning the datasets at their median values. The lower half of each split dataset (the ‘controls’) contained points below the median (e.g. very few daily deaths, or a negative day-to-day change in new deaths), while the upper half of each split dataset (the ‘cases’) contained points above the median (e.g. many daily deaths or a positive day-to-day change in new deaths). We then computed the average indoor conditions for cases and controls for the same range of time lags considered previously. This was performed for the new deaths (figure 4*a*), difference in new deaths (figure 4*b*), and percent change in new deaths (figure 4*c*) for each region of interest. We found that in the NH, more severe COVID-19 outbreaks were systematically associated with lower indoor RH. The estimated RH values dropped as time was shifted (representing the inclusion of colder/drier winter datapoints), but the pattern remained stable: more severe COVID-19 outbreaks were consistently characterized by lower indoor RH values. We observed the same pattern in the SH, with more severe COVID-19 outbreaks systematically associated with lower indoor RH. As time was shifted, RH values increased (representing the inclusion of data from the typically warmer/wetter summer), but the pattern still held: more severe COVID-19 outbreaks were consistently characterized by lower indoor RH values. Finally, when examining the Trop we found the opposite result: more severe COVID-19 outbreaks were systematically associated with higher indoor RH. However, we noted that, compared to the temperate zones, the tropical zones had much higher baseline humidity values. Therefore, it appeared that at low baseline RH values, an additional decrease in RH was associated with more severe COVID-19 outbreaks; conversely, at high baseline RH values, an additional increase in RH was associated with more severe COVID-19 outbreaks. Thus, greater COVID-19 spread and severity are reliably associated with more extreme indoor environmental conditions.

### 2.4. Odds of a worse COVID-19 outcome given indoor RH exposure

Existing scientific literature and guidelines from indoor air engineering both suggest that intermediate RH (approx. 40% to 60%) is best for optimal occupant health [46–48]. Through two different types of analysis (nonlinear modelling and case-control), we observed evidence that COVID-19 outcomes may be less severe at intermediate RH conditions. To explicitly examine the hypothetical ‘optimal’ RH range (40–60%), we discretized the continuous indoor RH measurements. The ‘extreme RH’ group contained all of the RH datapoints <40% or greater than 60%, while the ‘intermediate RH’ group contained all of the datapoints with RH falling between 40% and 60% (note that in electronic supplementary material, figures S7–S8, we examine the effect of changing the ‘optimal’ RH range assumption). The odds of a better or worse COVID-19 outcome were calculated based on exposure to either extreme RH or intermediate RH conditions. Finally, to compare the results we computed odds ratios and confidence intervals (figure 4*d*). Here, any value greater than 1.0 indicates that intermediate RH is associated with better COVID-19 outcomes (fewer new deaths, negative day-to-day change in new deaths), while any value less than 1.0 indicates that extreme RH is associated with better outcomes. Given the similar results across time lags, we present common odds ratios after pooling the time-lagged results (full figures and tables are in electronic supplementary material, figure S32). The estimated odds ratios and lower 95% confidence bounds were greater than 1 for new deaths, the day-to-day difference and percent change in new deaths (and for new cases, electronic supplementary material, figure S32). Critically, this result is consistent across all three regions. Thus, intermediate indoor RH is associated with epidemic deceleration and decreased mortality, while extreme indoor humidity (either low RH or high RH) is associated with epidemic acceleration and increased mortality. The collapse of the regionally variable results into a single trend suggests that intermediate indoor RH could potentially reduce the spread and severity of COVID-19 disease.

### 2.5. Assessing robustness and sensitivity to other factors

**Figure 5.**
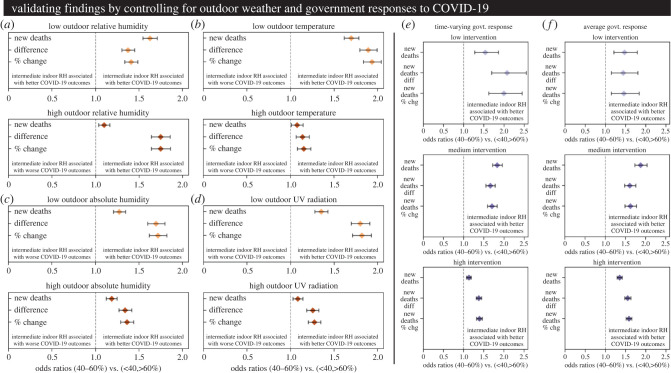
The association between intermediate indoor RH and better COVID-19 outcomes is robust. (*a*) Common ORs and 95% CIs created after stratifying the dataset by outdoor RH. (*b*) Common ORs and 95% CIs created after stratifying the dataset by outdoor temperature. (*c*) Common ORs and 95% CIs created after stratifying the dataset by outdoor absolute humidity. (*d*) Common ORs and 95% CIs created after stratifying the dataset by outdoor UV radiation. (*e*) Common ORs and 95% CIs created after stratifying the dataset by dynamic government responses (the overall government response stringency calculated by the Oxford COVID-19 Government Response Tracker OxCGRT group). The time lag between reported government response stringency and COVID-19 outcome was matched to the time lag between indoor RH and COVID-19 outcome to account for any dynamic effects (full results in electronic supplementary material figures S24–S25). (*f*) Common ORs and 95% CIs created after stratifying the dataset by mean government responses (average value calculated for each country over the timespan from normalized *t*_0_ to *t*_120_).

Since previous reports have focused on outdoor weather variables, it was important to test whether the results of our study held after controlling for differences in external weather conditions. Here, we stratified the global dataset by outdoor RH, temperature, outdoor absolute humidity (AH) or outdoor ultraviolet radiation (UV). The global dataset was partitioned into upper and lower halves using the median value of a given outdoor weather variable, and a series of common odds ratios were calculated for indoor RH exposure for each stratum. For outdoor RH (figure 5*a*), we found that intermediate indoor RH was significantly associated with better COVID-19 outcomes at both low and high outdoor RH conditions for all the COVID-19 metrics analysed. For outdoor temperature (figure 5*b*), we found that intermediate indoor RH was strongly associated with better COVID-19 outcomes at low temperature. This relationship still held at high outdoor temperature, though the magnitude of the association was much smaller. For outdoor AH (figure 5*c*) and outdoor UV (figure 5*d*), we found that intermediate indoor RH was again associated with better COVID-19 outcomes across all outdoor conditions. Like temperature, the magnitude was smaller for the high AH and high UV conditions, but all of the odds ratios and 95% confidence range for each of the outbreak metrics were greater than 1. The consistency across different AH levels is particularly interesting, as it shows that the RH association holds even when the absolute moisture content varies greatly, pointing to the physical processes known to be governed by RH (evaporation and condensation). In electronic supplementary material (figures S9–S12), we repeated our analytical workflow with additional outdoor weather variables as the predictor/treatment, and failed to find a robust and conserved trend (except for UV). Therefore, the association between indoor RH and COVID-19 holds whether regions are exposed to low or high outdoor RH, temperature, AH or UV. This supports the notion that indoor RH could potentially modulate the spread and severity of COVID-19 independent of the external weather conditions. These findings point to physical processes well known to be governed by RH, namely evaporation and condensation.

Finally, we further validated our results by controlling for widely varying government policies and interventions for COVID-19. Here, we stratified the global dataset according to the overall government response stringency (as computed by the OxCGRT team) [51]. This aggregate metric attempts to capture the breadth and depth of a government's response to coronavirus by combining various policies and government actions into a single daily measurement. We thus aimed to test whether the indoor RH relationship held after controlling for differing national responses to COVID-19 (detailed results can be found in electronic supplementary material, figures S24–S25). Since governments modified their strategies throughout the pandemic, we first stratified data by the time-varying government response stringency (figure 5*e*). We broke the dataset into three equally spaced bins representing ‘Low’, ‘Medium’ and ‘High’ government response stringencies. The time-lagged government responses were paired with the time-lagged indoor RH and common odds ratios were again calculated. We found that even when controlling for the dynamic government responses, the relationship between indoor RH and outbreak severity was conserved. Intermediate RH values (40% to 60%) were associated with better COVID-19 outcomes whether measured by new deaths, the difference in new deaths, or the percent change in new deaths. This trend held whether the government policies were of Low, Medium or High stringency (though the magnitude was smallest for the highest government stringency). One drawback to examining the time-varying stringency is that a country with currently strict government policies could still experience severe COVID-19 outbreaks because of much weaker guidelines earlier in their pandemic. Therefore, as a complementary validation we also stratified the data according to the time-averaged government response stringency (mean national responses were computed for each country across the normalized outbreak timeframe from *t*_0_ to *t*_120_). Again, we found that intermediate RH was associated with better COVID-19 outcomes across all metrics for Low, Medium, and High average government response stringency (figure 5*f*). Taken together, these results suggest that indoor RH could affect the spread and severity of COVID-19 independent of the relevant government interventions and public health guidelines for a given country.

For additional analyses, we direct the reader to extensive electronic supplementary material, where we present the results from a wide range of sensitivity analyses and robustness checks. In §1 (electronic supplementary material, figures S1–S6), we quantify how changing the assumed thermal comfort zone (sweeping from 19°C to 25°C) modified the resulting indoor RH and ultimately the observed relationships between indoor RH and COVID-19. In extended online material we further analyse from 18°C down to 12°C to assess the implications of lower assumed indoor temperatures. In §2 (electronic supplementary material, figures S7–S8), we explore how changing the assumed optimal RH range (in the main text, 40% to 60%) modified the observed relationships between indoor RH and COVID-19. In §3 (electronic supplementary material, figures S9–S12), we quantify the relationships between outdoor weather variables and COVID-19 using the same analytical approaches from the main text. In §4 (electronic supplementary material, figures S13–S20), we performed a full analysis of indoor and outdoor data collected in various locations during cold months and provide a comprehensive validation of our indoor RH extrapolation workflow used here. In §5 (electronic supplementary material, figures S21–S23), we examine further regional stratification in the tropical regions of the globe. In §6 (electronic supplementary material, figures S24–S25), we delved further into varying national policies and investigated more granular stratifications by government response. In §7 (electronic supplementary material, figures S26–S29), we analyse the robustness of the results after controlling for outdoor weather variables. In §8 (electronic supplementary material, figures S30–S32), we completed a more comprehensive study partially cut from the main text for brevity. This includes the results from our global-level analysis as well as the results we obtained when using daily COVID-19 cases as a dependent variable (in addition to the death-related metrics we displayed here in the main text). In §9 (electronic supplementary material, figures S33–S34), we retrieved and processed new datasets to cover the period of January 2020 to January 2021 and obtain updated and extended COVID-19 statistics and meteorological data from 155 countries. We repeated the analysis from the main text to confirm the validity of our main conclusions on these larger datasets that included more countries and more datapoints per country. In §10 (electronic supplementary material, figures S35–S36), we quantify how parameter modifications in the regression algorithms altered the fitted models. In §11 (electronic supplementary material, figures S37–S39), we quantify the effects of modifying the available analytical window of the normalized outbreak timeline. In §12 (electronic supplementary material, figures S40–S41), we quantify the sensitivity of the results and conclusions on the data pre-processing approach. And in §13 (electronic supplementary material, tables S1–S8), we provide results from statistical hypothesis tests as well as the bounds of the quantized data used in figures 4 and 5 for any interested readers. Overall, from these extended robustness tests, we found the relationships presented in the main text to be robust even when controlling for a number of other factors and when using many different variations of the methodology, showing that indoor RH is consistently associated with COVID-19 spread and severity. For brevity, a curated subset of the analysis is included in the electronic supplementary material.

## 3. Discussion

Inter-country variability in both outbreak magnitude and reporting conditions produced a complex and noisy dataset that precluded simple description or modelling [49]. Therefore, we believed it was critical to explicitly account for non-uniform outbreak start dates, non-uniform outbreak lengths, temporal trend in cumulative COVID-19 metrics, and potential time-lagged dependencies. Interestingly, the same general patterns tended to persist even when differing time lags were considered and when different data treatments were applied. The stability of those trends may be a function of the high-level nature of the study, where data aggregation masks lower-level variability but reveals more statistically reliable structure within the data [49].

The results reported here do reveal a systematic association between COVID-19 and indoor RH. Regarding underlying mechanisms, any causal relationship between RH and respiratory viral disease is expected to be complex and multifaceted, involving rich biophysical and fluid physcis processes and physiology modulated by environmental factors [11]. From a physiological standpoint, dry conditions were reported to damage nasal and tracheal epithelium, decrease mucociliary clearance and change mucin production, all of which can alter airway mucosal function [52–54]. Dry conditions were reported to also potentially impair tissue repair and increase viral disease burden and mortality [44]. From a biophysical standpoint, we expect viral viability to depend strongly on ambient conditions, with reported viral persistence following a nonlinear or U-shaped dependency on RH or temperature. Notably, intermediate RH led to higher observed decay in some cases, including for coronaviruses in animals [55–58]. Beyond viral stability, the size, evaporation and spread of respiratory droplets and aerosols also influence viral transmissibility with previous studies reporting heightened viral transmission at low or high RH levels [59,60]. Others have hypothesized that low RH favours airborne transmission by increasing the fraction of desiccated aerosols, while high RH would favour contact transmission by ensuring long-term hydration of respiratory emissions [61,62].

A number of questions remain open on the mechanisms by which RH and other indoor climate variables affect viruses in microscale respiratory droplets [4]. We here revealed an apparent association between indoor RH and COVID-19 at the global population scale, but additional ongoing work focuses on bridging macroscale and microscale results. On the one hand, follow-up top-down studies of population data could investigate the indoor RH hypothesis at higher spatial resolution to determine the robustness of the relationship as a function of scale; on the other hand, bottom-up biophysical experimental–theoretical investigations are needed to shed light on the multifaceted mechanisms and rationalize the inconsistent results so far reported [9,10,12].

Overall, these correlational results suggest that COVID-19 outbreaks could potentially be modulated by indoor RH conditions. Therefore, indoor RH optimization represents a promising potential target for public health intervention [63]. Such efforts are of particular importance as airborne transmission is involved [64]. Unlike seasonal weather patterns, indoor climate is something that can feasibly be controlled by humans (e.g. by occupants or building managers). Compared to highly disruptive measures (e.g. closing society), maintaining optimal indoor RH would be much less disruptive and costly, as an adjunct to other containment measures. Unlike measures that depend on individual compliance (e.g. masking or hand-washing), indoor RH optimization would achieve high compliance because all occupants of a common indoor space would be exposed to similar ambient conditions. Compared to the long timelines and high costs of vaccine production and distribution, humidity control systems could potentially be implemented more quickly and cheaply in certain indoor settings. However, we want to explicitly state that the aforementioned public health measures (and others) are critical and necessary components of a holistic COVID-19 response [65,66]. Many studies have already demonstrated the vital importance of non-pharmaceutical interventions and COVID-19 mitigation policies [67,68]. Environmental conditions cannot and will not halt the spread of the virus *alone*. Further, additional variables such as ventilation rate can affect the indoor spread and severity of respiratory disease, and simultaneous optimization of humidity and ventilation may be needed. With continual resurgences of COVID-19 in many parts of the world, and with the prospect of more virulent strains, we propose that indoor RH optimization, maintained to be between 40% and 60%, could complement existing COVID-19 countermeasures and contribute to a multifaceted [69] effort to minimize indoor disease transmission.

## 4. Conclusion

We hypothesized that indoor rather than outdoor ambient conditions were the relevant factors to consider when studying respiratory viral outbreaks. In our study, we retrieved and rigorously processed an extensive global dataset of COVID-19 statistics and meteorological variables, and then extrapolated and validated indoor RH levels. Through time-series visualization, robust regression, case-control analysis and retrospective cohort analysis, we comprehensively explored the relationships between regional indoor RH and COVID-19 spread and severity. Overall, the data pointed toward a consistent and robust pattern: COVID-19 outcomes are less severe at intermediate indoor RH levels (between 40% and 60%) and more severe at extreme indoor RH levels. This association is found to be robust to variation in geographical region, outdoor weather conditions, and government responses, as well as to variations in the underlying methodology.

## 5. Material and methods

### 5.1. Retrieving global data

For this study, we aimed to accumulate a dataset with global coverage of the coronavirus outbreaks. Coronavirus statistics for each country were extracted from the JHU and ECDC datasets. Latitude and longitude values for each country's geographical centroid were used for pairing with meteorological data. Countries were selected for inclusion by applying a threshold of 50 confirmed COVID-19 deaths (or 100 confirmed deaths for the follow-up validation dataset):

Cumulative deaths≥50.

This stringent filter served to exclude any countries that were too early in their outbreak time course to adequately capture any relationship between environmental variables and COVID-19 outbreak severity. Because each country's outbreak started on different dates throughout the year, we normalized the unique outbreak timecourses by a cumulative death threshold. This operation served to collapse all of the outbreaks on a common time scale for better comparison. For each country, the date where the cumulative death total was greater than or equal to five deaths was set as the reference time zero. Dates before zero were assigned negative integers (counting down to zero) and dates after zero were assigned positive integers (counting up from zero):

$$\mathrm{Reference}\;t_{0}=\mathrm{Calendar}\;\mathrm{date}\;\mathrm{where}\;\mathrm{cumulative}\;\mathrm{deaths}\geq 5.$$

We retrieved global meteorological data from the ERA5 reanalysis dataset compiled by the European Centre for Medium-Range Weather Forecasts (ECMWF). Data were accessed through the Climate Data Store (CDS) API provided by the Copernicus Climate Change Service (C3S). Specifically, we accumulated measurements for 2-metre dewpoint and 2-metre temperature, at 06.00, 12.00 and 18.00 daily timepoints, at 0.5° × 0.5° spatial grid resolution, for each day from 22 January 2020 to 9 August 2020. The spatially and temporally resolved meteorological dataset was then matched to the coronavirus dataset according to the geographical and date time information accompanying each selected outbreak country. The three daily timepoints for each variable were averaged to get a single daily measurement for each country. Each country was paired with meteorological information from 35 days before their fifth cumulative death and up to the most recent date accessible in the downloaded weather dataset.

### 5.2. Computation of indoor environmental conditions

We computed the average indoor conditions for each country using the measured outdoor conditions variables and the established human thermal comfort zone. In some cases, the RH is given directly from measurements outdoors. In other cases, we compute it using reported outdoor 2-metre temperature and outdoor 2-metre dewpoint measurements, via the August–Roche–Magnus approximation (equation (5.1)) [70,71]:

5.1 $$\mathrm{RH}=100\times \frac{\exp(17.625\times T_{D}/243.04+T_{D})}{\exp(17.625\times T/243.04+T)},$$

where RH is the relative humidity in %, *T*_D_ is the outdoor dewpoint in °C and *T* is the outdoor temperature in °C. Then, the outdoor absolute humidity can be calculated (equation (5.2)) [70]:

5.2 $$\mathrm{AH}=6.112\times \frac{\exp(17.625\times T/243.04+T)\times \mathrm{RH}\times 2.1674}{273.16+T},$$

where AH is the outdoor absolute humidity in g m^−3^. Alternatively, we could first extrapolate the outdoor vapour pressure at the dewpoint temperature (equation (5.3)) and then compute the outdoor absolute humidity (equation (5.4)) [72]:

5.3 $$e_{d}=0.6108\times \exp\left(\frac{17.27\times T_{d}}{T_{d}+237.3}\right)$$

and

5.4 $${\mathrm{AH}}_{\mathrm{alt}}=\frac{2165\times e_{d}}{T+273.16},$$

where *e*_d_ is the outdoor vapour pressure in kPa. A comparison of the first method (equations (5.1) and (5.2)) and the second method (equations (5.3) and (5.4)) for AH computation showed a root mean squared error of 0.016 g m^−3^ across a *T* sweep of −10 to 40°C and a *T*_d_ sweep of −10 to 40°C (2500 calculations total), so we concluded the first method (equations (5.1) and (5.2)) was valid and we used that for remaining computations.

Finally, the outdoor temperature, outdoor AH and approximate human thermal comfort zone were used to estimate the indoor RH conditions by comparing actual vapour density with saturation vapour density (VD_sat_) [73]. In the results presented in the main text, we assumed an indoor comfort temperature of 21°C. However, in electronic supplementary material (figures S1–S6), we investigated the effects of modifying the assumed thermal comfort zone, and we demonstrated that the study's main conclusions (relationship between RH and COVID-19 spread and severity) are valid across an indoor temperature range of 19°C to 25°C.

If the outdoor temperature (*T*_o_) is less than the human thermal comfort temperature (*T*_c_), we assume the indoor space is heated and compute VD_sat_ in g m^−3^ using equation (5.5):

5.5 $${\mathrm{VD}}_{\mathrm{sat}}=5.02+0.32(T_{c})+8.18\times 10^{-3}(T_{c}^{2})+3.12\times 10^{-4}(T_{c}^{3}).$$

If the outdoor temperature (*T*_o_) is greater than or equal to the human thermal comfort temperature (*T*_c_), we assume the indoor space is not heated and compute VD_sat_ in g m^−3^ using equation (5.6):

5.6 $${\mathrm{VD}}_{\mathrm{sat}}=5.02+0.32(T_{o})+8.18\times 10^{-3}(T_{o}^{2})+3.12\times 10^{-4}(T_{o}^{3}).$$

RH can then be computed by dividing the actual vapour density in the air by the saturation vapour density of the air (VD_sat_). Since AH (in g m^−3^) is equivalent to the actual vapour density, the indoor relative humidity (RH_indoor_) can be computed via equation (5.7):

5.7 $${\mathrm{RH}}_{\mathrm{indoor}}=\frac{\mathrm{AH}}{{\mathrm{VD}}_{\mathrm{sat}}}.$$

### 5.3. Validating the indoor climate computation: from approximation to experiments

The August–Roche–Magnus approximation allows for very good estimation of saturation vapour pressure as a function of the ambient temperature. This Magnus formula is an approximation of the Clausius–Clapeyron relation and is used frequently in meteorology [74]. To validate the approximations, we compared the results from the August–Roche–Magnus approximation to the results from the analytical solution to the Clausius–Clapeyron relation (equation (5.8)) with Clausius–Clapeyron linking water vapour near typical atmospheric conditions:

5.8 $$\frac{de_{s}}{dT}=\frac{L(T)\times e_{s}}{R\times T^{2}},$$

where *e*_s_ is the saturation vapour pressure, *L*(*T*) is the specific latent heat of evaporation of water, *R* is the gas constant of water vapour and *T* is the absolute temperature. If we assume latent heat *L*(*T*) is approximately constant at these atmospheric conditions we obtain equation (5.9):

5.9 $$\frac{de_{s}}{dT}=\left(\frac{L}{R}\right)\frac{e_{s}}{T^{2}}.$$

By separation of variables we obtain the following relation (equation (5.10)):

5.10 $$\frac{1}{e_{s}}de_{s}=\left(\frac{L}{R}\right)\frac{1}{T^{2}}dT,$$

which can be integrated as follows (equation (5.11)):

5.11 $$\int_{e_{0}}^{e_{s}}\frac{1}{e_{s}}\;de_{s}=\frac{L}{R}\int_{T_{0}}^{T}\frac{1}{T^{2}}\;dT,$$

yielding the following relation (equation (5.12)):

5.12 $$\ln(e_{s})-\ln(e_{0})=\frac{L}{R}\left(-\frac{1}{T}--\frac{1}{T_{0}}\right).$$

This can be rearranged into (equation (5.13))

5.13 $$\ln\left(\frac{e_{s}}{e_{0}}\right)=\frac{L}{R}\left(\frac{1}{T_{0}}-\frac{1}{T}\right).$$

Exponentiating and isolating *e*_s_ (equation (5.14)):

5.14 $$e_{s}=e_{0}\exp\left(\frac{L}{R}\left(\frac{1}{T_{0}}-\frac{1}{T}\right)\right).$$

We use *e*_0_ = 611.65 Pa, *L* = 2 500 800 J kg^−1^, *R* = 461.524 J kg K^−1^, *T*_o_ = 273.15 K for comparison of results from the analytical solution to the Clausius–Clapeyron relationship to results from the August–Roche–Magnus approximation. In electronic supplementary material (figures S13–S14), we compare (1) saturation vapour pressure estimations across a wide range of temperatures, (2) RH estimations across a wide range of temperatures and dewpoints and (3) the absolute error in RH estimations across a wide range of temperatures and dewpoints. Overall, we found that the mean absolute error in RH between the Clausius–Clapeyron analytical solution and the August–Roche–Magnus approximation was 0.26 ± 0.31%.

After validating the approximation accuracy, we then validated the indoor climate extrapolation workflow. First, we used literature values from a study conducted in March and April of 2020 [50]. The authors measured the indoor temperature and RH at three different teaching hospitals in Boston, MA. Second, we used our own data collected from sensors placed in different types of buildings over different timeframes in autumn–winter 2020–2021. To validate our extrapolation workflow, we retrieved outdoor weather data from the corresponding locations during the same time periods and fed the data into our indoor climate approximation pipeline. In electronic supplementary material (figures S15–S20), we compare (1) outdoor temperature and humidity timeseries, (2) outdoor and indoor temperature and humidity and (3) measured indoor RH and extrapolated indoor RH for each of the validation datasets. Overall, we find that our approximations of the indoor conditions are accurate on average, and are much closer to the actual ambient conditions that occupants were experiencing than what would be assumed if only the outdoor weather was considered.

### 5.4. Regional data aggregation

The retrieved and processed dataset contained data from 121 different countries around the globe. To better explore regional patterns, we defined Northern Hemisphere countries (*n* = 67) as those in the temperate zone with a latitude north of the Tropic of Cancer (lat > 23.5°), Southern Hemisphere countries (*n* = 4) as those in the temperate zone with a latitude south of the Tropic of Capricorn (lat < −23.5°), and the Tropics countries (*n* = 50) as those in the tropical zone with latitude between the Tropic of Cancer and the Tropic of Capricorn (−23.5° < lat < 23.5°). Datapoints were aggregated by region to facilitate comparisons of temperate and tropical regions. Further sub-division is possible in the Tropics, and we present the effects of splitting into northern and southern Tropics in electronic supplementary material (figures S21–S23)

### 5.5. COVID-19 outbreak metrics

From the literature, it is clear that the COVID-19 transmission–infection–resolution timecourse can be highly variable. Thus, reliable prediction of COVID-19 outcomes at a granular, day-to-day timescale becomes highly challenging. Additionally, all of the continuous metrics showed a high degree of day-to-day variability. We therefore applied a 7-day centred rolling average to both the inputs and outputs of our dataset in order to smooth high-frequency noise (day-to-day variability in measurements) while preserving the underlying signal (epidemic dynamics occurring on a longer timescale).

Cumulative metrics like total confirmed cases or total confirmed deaths could mask important information about the underlying evolution of the individual epidemics because of country-to-country heterogeneity in outbreak start date. Purported weather-dependent trends in COVID-19 severity could simply be the result of selection bias, with a non-random distribution of outbreaks around the globe at a given timepoint. To account for this, we used only the first 120 days of each country's normalized outbreak in the downstream regressions and statistical comparisons. In electronic supplementary material (figure S37), we show that at *t* = *t*_120_ = 120 days, 75% of the total countries in the dataset are still included. However, this number declines precipitously as the normalized timescale increases, meaning that the earliest countries will begin to exert a stronger and stronger influence on the results of the analysis. The 120-day cutoff served to reduce the impact of these countries and provide a more accurate comparison across the global dataset. In electronic supplementary material (figures S38–S39) we examine the consequences of shrinking or expanding this temporal analytical window. To further de-weight the importance of countries that simply experienced an earlier outbreak starting date, we focused on non-cumulative metrics. Specifically, we examined daily new COVID-19 deaths (equation (5.15)), the day-to-day difference in new COVID-19 deaths (equation (5.16)), and the day-to-day percent change in new COVID-19 deaths (equation (5.17)). These measurements would allow us to more precisely capture relationships between time-varying ambient conditions and time-varying epidemic curves.

5.15 $$\mathrm{New}\;\mathrm{Deaths}=\frac{\mathrm{Total}\;\mathrm{Deaths}(t)-\mathrm{Total}\;\mathrm{Deaths}(t_{-1})}{t-t_{-1}},$$

5.16 $$\mathrm{New}\;\mathrm{Deaths}\;\mathrm{Difference}=\frac{\mathrm{New}\;\mathrm{Deaths}(t)-\mathrm{New}\;\mathrm{Deaths}(t_{-1})}{t-t_{-1}}$$

5.17 $$\mathrm{and}\;\mathrm{New}\;\mathrm{Deaths}\;\text{\%}\mathrm{Change}=\frac{\mathrm{New}\;\mathrm{Deaths}(t)-\mathrm{New}\;\mathrm{Deaths}(t_{-1})}{\mathrm{New}\;\mathrm{Deaths}(t_{-1})(t-t_{-1})}\times 100.$$

The serial differencing operations served to reduce the trend component of the cumulative COVID-19 outbreak signal (which can be observed in electronic supplementary material, figure S30). The % change (equation (5.17)) was analogous to the second difference (equation (5.16)), but it also eliminated the scale dependency of the metric. Because of the well-documented variability in testing infrastructure and testing requirements, we chose to focus most of our analysis on the aforementioned COVID-19 death statistics rather than on cases (which can depend strongly on testing). However, we have included new confirmed COVID-19 cases as an additional metric of comparison in electronic supplementary material (figures S31–S32).

### 5.6. Regression analysis

Given the temporal evolution of the climate measurements and COVID-19 outbreak metrics, correlations and regressions were computed on time-shifted (lagged) versions of the dataset, from 0 days of lag all the way up to 28 days of lag [75]. Given the potential for regional heterogeneity, global correlations and regressions were compared to those found within the NH, SH or Trop regions.

Robust linear regressions were performed to determine if any linear relationship could be captured once the effect of outliers in the dataset had been reduced [76]. We specifically implemented an iteratively re-weighted least-squares approach with Huber's *T* weighting function. In electronic supplementary material (figure S36), we demonstrate the difference between our robust linear model and an ordinary least squares model. We also compared the effect of different robust criterion functions for de-weighting outliers (*M* estimation functions = Huber's *T* function, Ramsay's *E* function, Andrew's wave function, trimmed mean function, Hampel function and Tukey's biweight function).

Non-parametric LOWESS fitting of the same data was performed to see if any nonlinear relationships could be recovered when the model form was not specified [77]. We used a robust method that first estimated outputs with a tricube-weighted local linear regression. Then, the method iteratively performed additional locally weighted linear regressions with the weights further modified by the LOWESS bisquare function of the residuals from the previous fit, thereby de-weighting the effect of datapoints with large residuals. Default values were used for the modelling parameters, with 2/3 of the data used when estimating each dependent variable and 3 residual-based reweightings performed for each fit. In electronic supplementary material, we investigate the effect of modifying these default parameters.

### 5.7. Quantization of the dataset

To facilitate relative comparisons and further reduce the impact of outliers, the continuous COVID-19 outbreak measurements were discretized into quantiles by breaking the dataset at the median outbreak value [78]. As a note, some values (like zero) were highly frequent in the dataset (e.g. many countries have spans of data reporting that do not contain any new COVID-19 deaths). This relative over-representation of a single value led to quantiles with differing numbers of samples, since a frequently repeated value cannot be present in both bins. Nevertheless, we found the quantization procedure reliably produced approximately equally distributed bins. Datapoints were split according to the median value, which therefore partitioned the dataset into upper and lower halves. After quantization, the dataset could then be effectively grouped by relative COVID-19 severity. Here, the lower half of a given quantized COVID-19 metric would represent a relatively ‘better’ outbreak (fewer new deaths, smaller or even negative difference in new deaths, smaller or even negative percent change in new deaths). Conversely, the upper half of a given quantized COVID-19 metric would represent a relatively ‘worse’ outbreak (more new deaths, larger positive difference in new deaths, larger positive percent change in new deaths).

Like the COVID-19 outputs, the meteorological inputs can also be discretized. Based upon previous literature [40–42] and the results from our analysis, we chose to apply a custom discretization method. Rather than splitting the data at the median value, we split the data according to user-supplied bin ranges. Specifically, the indoor RH was split into the values falling between 40% and 60% and any value falling outside of that range (<40% or >60%) [43–45]. After quantization, the dataset could then be effectively grouped by indoor climate, where the locations exposed to the hypothetical optimal ambient conditions (40–60% RH) could act as a ‘treatment group’, while the locations not exposed to the hypothetical optimal ambient conditions (<40% or >60% RH) could act as a ‘control’ or ‘untreated group’. (Note: we consider alternative ‘optimal’ RH ranges and examine their effects on the final results in electronic supplementary material, figures S7–S8.)

### 5.8. Calculation of odds ratios

The discretized measurements were paired to create a 2 × 2 contingency table (table 1). One axis represents the exposure to optimal indoor conditions (between 40% and 60% or outside of 40–60%), and the other axis represents the COVID-19 outbreak severity (lower half or upper half of the dataset).

**Table 1.** 2 × 2 contingency table to compute odds ratios.

generalized contingency table		indoor RH exposure	
		(<40% or >60%)	(40–60%)
COVID-19 outcomes	more severe	no. datapoints (*A*)	no. datapoints (*B*)
	less severe	no. datapoints (*C*)	no. datapoints (*D*)

The odds of a worse COVID-19 outcome (e.g. odds of having more new deaths) can be calculated by comparing the frequency of belonging to either the lower or upper half of the quantized outbreak metric (equation (5.18)):

5.18 $$\mathrm{Odds}=\frac{\mathrm{No}.\;\mathrm{more}\;\mathrm{severe}\;\mathrm{outcomes}}{\mathrm{No}.\;\mathrm{less}\;\mathrm{severe}\;\mathrm{outcomes}}=\frac{A}{C}\;\mathrm{or}\;=\frac{B}{D}.$$

The odds ratio (equation (5.19)) could then be calculated by comparing the odds of a worse outcome for the unexposed group (less than 40% or greater than 60%) to the odds of a worse outcome for the exposed group (40–60%):

5.19 $$\mathrm{Odds}\;\mathrm{ratio}=\frac{{\mathrm{Odds}}_{\left(<40\text{\%}\;\mathrm{or}\;>60\text{\%}\right)}}{{\mathrm{Odds}}_{\left(40-60\text{\%}\right)}}=\frac{A/C}{B/D}.$$

When controlling for outdoor weather conditions, we first pooled all of the regional datasets to again obtain a global dataset. Then, rather than stratifying by regional identifier, we stratified by the median outdoor weather measurements. Specifically, we broke the continuous outdoor weather measurements into two equally distributed bins. Odds ratios and confidence intervals for indoor RH exposure and COVID-19 outbreak severity were computed for each strata for each outdoor weather variable (outdoor RH, outdoor temperature, outdoor absolute humidity and outdoor UV radiation). Additional results related to outdoor weather conditions can be found in electronic supplementary material, figures S9–S12 and S26–S29 (analysing COVID-19 versus outdoor weather variables and analysing indoor RH after controlling for outdoor weather variables).

When controlling for government response stringency, we first pooled all of the regional datasets to again obtain a global dataset. Then, rather than stratifying by regional identifier, we stratified by discretized government response stringency. Specifically, we broke the continuous stringency metric (ranging from 0 to 100) into three equally spaced bins. Then, we compared the effects of stratifying by time-varying government stringency (incorporating time lags) or stratifying by static government stringency (computing the average government response over the entire normalized outbreak timecourse for each country). Odds ratios and confidence intervals for indoor RH exposure and COVID-19 outbreak severity were computed for each strata for each government response stringency level (Low, Medium and High).

### 5.9. Software and packages

Data analysis was conducted using Python v. 3.6.9. All scripts, datasets, and IPython notebooks were stored in a Github repository. To allow other researchers to easily view, run and modify the notebooks, all data visualization and analysis were conducted in the free Google Colaboratory cloud-based Python environment. Python libraries (and library version numbers) used for data visualization and analysis include matplotlib 3.2.2, numpy 1.18.5, pandas 1.0.5, scikitlearn 0.22.2, scipy 1.4.1, seaborn 0.10.1 and statsmodels 0.10.2.

### 5.10. Disclaimers and caveats

Like other COVID-19 studies, any statistical analysis is limited by the available epidemiological data. Coronavirus statistics can vary in their reliability for a number of causes. First, government testing and healthcare infrastructures, as well as government data-reporting policies, can vary significantly from country to country. Second, it is accepted that current data collection methods do not capture every true positive case. Likewise, the true COVID-19-related mortality is probably higher than the number of confirmed COVID-19 deaths. Finally, any attempt to draw conclusions based upon the longitudinal, time-varying outbreak metrics can be confounded by the highly variable time lags between COVID-19 transmission event, infection progression and symptom appearance and downstream death or recovery outcome. To mitigate these effects, we performed a number of additional robustness tests and sensitivity analyses via systematic methodology comparison. Here we chose to ignore air conditioning (cooling hot external air) and only consider indoor heating (warming cold external air) for this analysis as most households and indoor spaces worldwide do not in fact use air conditioning. Although developed regions and developing regions may have different methods for heating (natural gas or electric infrastructure versus solid biomass combustion), indoor heating consumes a significant portion of the total energy expenditure during the heating season in both developed and developing regions and thus indoor heating is much more prevalent globally than indoor cooling. In this study, we chose to specifically explore the potential association between extrapolated indoor climate and COVID-19 outbreak severity. The results above suggest that such a link exists. The underlying mechanism linking the two is not however revealed by this analysis. Relevant factors rooted in the biophysics of virus persistence and fluid physics involved coupled with the biology would be the source of the true causal effect on COVID-19 outbreaks [4,53].

### 5.11. Statistical analysis

For all timeseries visualizations, fitted model predictions, and bar charts bootstrapped 95% confidence intervals were constructed around each mean estimate. As described above, the robust linear modelling was performed with an iteratively re-weighted least-squares approach with Huber's *t* weighting function, and the robust nonlinear modelling was performed with a tricube-weighted local linear regression that was iteratively re-weighted by the LOWESS bisquare function of the residuals from the previous fit. For the bar charts in figure 4, the Mann–Whitney *U* test was used to compare groups given their non-normal data distributions. For the pooled odds ratios in figures 4 and 5, the Breslow–Day test was used to test for equivalence of individual odds ratios, and the Mantel–Haenszel test was used to test for an odds ratio greater than 1.0. For all hypothesis tests conducted, the strict Bonferroni correction was applied to account for multiple comparisons.

## Data Availability

All datasets, codes, and analysis notebooks can be accessed in the following GitHub repository: https://github.com/connor-verheyen/COVID19_IndoorRH or associated Zenodo repository: https://doi.org/10.5281/zenodo.7195705 [79]. When using any part of this repository, cite C. A. Verheyen and L. Bourouiba (2022) Associations between indoor relative humidity and global COVID-19 outcomes. Journal of the Royal Society Interface, doi:10.1098/rsif.2021.0865. The data are provided in electronic supplementary material [80].
